# N-glycosylation stabilizes CD155 to evade NK cell surveillance in colorectal cancer

**DOI:** 10.1038/s41420-026-03302-9

**Published:** 2026-08-13

**Authors:** Jiahe Xu, Dongze Zhang, Kexi Yang, Jiao Xue, Yongqiang Su, Jiayu Wang, Songbing Qin

**Affiliations:** 1 Department of Radiation Oncology, First Affiliated Hospital of Soochow University, Suzhou, China; 2 Department of Gastroenterology, The First Affiliated Hospital of Soochow University, Suzhou, China; 3 Jiangsu Institute of Clinical Immunology, The First Affiliated Hospital of Soochow University, Suzhou, China; 4 Gastroenterology Department, Nantong First People’s Hospital, Southeast University, Nantong, Jiangsu China

**Keywords:** Tumour-suppressor proteins, Glycosylation

## Abstract

The majority of patients with colorectal cancer (CRC) fail to respond to anti-PD-1/PD-L1 immunotherapy, highlighting an urgent need for alternative immunotherapeutic targets. The immunosuppressive ligand CD155 potently dampens natural killer (NK) cell cytotoxicity via inhibitory receptors, but how CRC cells sustain high CD155 surface levels remains elusive. Here, we identify aberrant CD155 N-glycosylation as a critical driver of CRC immune evasion. We find that the glycosyltransferase STT3A contributes to N-glycosylation of CD155. This modification is essential for maintaining CD155 protein stability, thereby sustaining its surface expression to suppress NK cell-mediated antitumor immunity. Consistent with a role for N-glycosylation in maintaining CD155 expression, pharmacological inhibition of oligosaccharyltransferase activity with NGI-1 promotes CD155 turnover, effectively abrogating its immunosuppressive function and enhancing NK cell-mediated tumor clearance in vivo. Collectively, these findings implicate the STT3A-CD155 glycosylation axis as a therapeutic vulnerability, providing a mechanistic rationale for reprogramming the immunosuppressive CRC microenvironment.

## Introduction

Colorectal cancer (CRC) is the third most common malignancy and the second leading cause of cancer-related mortality worldwide [1]. Immune checkpoint blockade (ICB) has fundamentally revolutionized cancer immunotherapy by restoring the antitumor functions of immune effector cells—including CD8^+^ T cells and natural killer (NK) cells—that are frequently rendered dysfunctional within the immunosuppressive tumor microenvironment (TME) [2, 3]. In patients with advanced CRC, microsatellite instability‑high or deficient mismatch repair (MSI‑H/dMMR) status predicts favorable responses to anti‑PD‑1 therapy [4, 5]. However, up to half of CRC patients have microsatellite stable or proficient mismatch repair (MSS/pMMR) tumors, which derive minimal benefit from ICB monotherapy [6–8]. Moreover, even among initial responders, acquired resistance frequently leads to disease progression [7, 9]. These clinical limitations highlight the urgent need to identify and target additional immune regulatory pathways to improve response rates and the therapeutic durability of immunotherapy in CRC.

Among the emerging immune checkpoint targets, the poliovirus receptor (PVR, also known as CD155) has attracted considerable attention. CD155 is a transmembrane glycoprotein belonging to the immunoglobulin (Ig) superfamily, which is primarily expressed on antigen-presenting cells and tumor cells [10–12]. Importantly, CD155 expression is significantly elevated in advanced-stage tumors compared to early-stage disease and inversely correlates with patient survival [12–14]. In addition to promoting tumor cell proliferation and migration [15–17], CD155 drives immune escape by engaging both host and tumor-intrinsic pathways, contributing to its multifaceted role in tumor progression [10, 18–20]. CD155 engages several co-stimulatory and co-inhibitory receptors on immune cells, including CD96, TIGIT, and CD226 (DNAM-1) [21–23]. Notably, CD155 has a higher affinity for TIGIT than it does the activating receptor CD226 [23], such that CD155 overexpression on tumor cells decisively shifts the signaling balance toward immune suppression. The functional consequences of this inhibitory axis are particularly pronounced in NK cells. When bound to TIGIT, CD155 can directly inhibit NK cytotoxicity in an ITIM motif-dependent manner [24]. Indeed, TIGIT-positive NK cells—despite exhibiting a mature and highly cytotoxic profile—are functionally suppressed by CD155-overexpressing tumor cells [25]. However, the precise immunoregulatory role of CD96 remains incompletely defined. While CD96 possesses an ITIM motif typically associated with the transmission of inhibitory signals, it simultaneously contains a YXXM motif, which is implicated in the activation of downstream signaling pathways [26, 27]. Thus, CD155 plays a key role in immune escape, but the mechanisms by which tumor cells maintain high levels of cell-surface CD155 remain incompletely understood.

Targeting post-translational modifications (PTMs), particularly N-glycosylation, represents an emerging and promising strategy to disrupt such immune-inhibitory axes [28, 29]. Immune checkpoint molecules (ICMs) are frequently modified by complex glycans that precisely regulate their synthesis, subcellular localization, degradation and function. N-glycosylation is initiated in the lumen of the endoplasmic reticulum and is catalyzed by the oligosaccharyltransferase (OST) complex, which contains two catalytic subunit paralogs, STT3A and STT3B [28, 30]. Accumulating evidence indicates that PD-L1 glycosylation contributes to maintaining its protein stability and interaction with PD-1 on immune cells. Targeting PD-L1 glycosylation disrupts the PD-1/PD-L1 interaction, thereby enhancing T-cell cytotoxicity and improving antitumor immune responses [31–33]. Similarly, targeting the glycosylation of other ICMs, such as B7-H4 and B7-H3, has also been demonstrated to improve immunotherapy outcomes [34–37]. CD155 is also heavily glycosylated, and its glycosylation has been reported to be critical for interactions with CD226 and TIGIT on NK cells, thereby influencing NK cell activity in vitro [38]. However, the molecular mechanisms underpinning CD155 glycosylation within the TME and the functional significance of this modification for antitumor immunity in vivo remain poorly understood.

In this study, we identify and characterize the N-glycosylation of CD155 in colorectal tumors and demonstrate that the OST catalytic subunit STT3A contributes to this modification. Functionally, STT3A-dependent glycosylation of CD155 promotes tumor immune evasion by suppressing NK cell-mediated antitumor activity. Notably, elevated co-expression of CD155 and STT3A is associated with poorer survival outcomes in CRC patients. Collectively, our findings suggest that targeting N-glycosylation of CD155 represents a promising strategy to enhance the efficacy of CRC therapy.

## Results

### CD155 is N-glycosylated in CRC cells

**Fig. 1:**
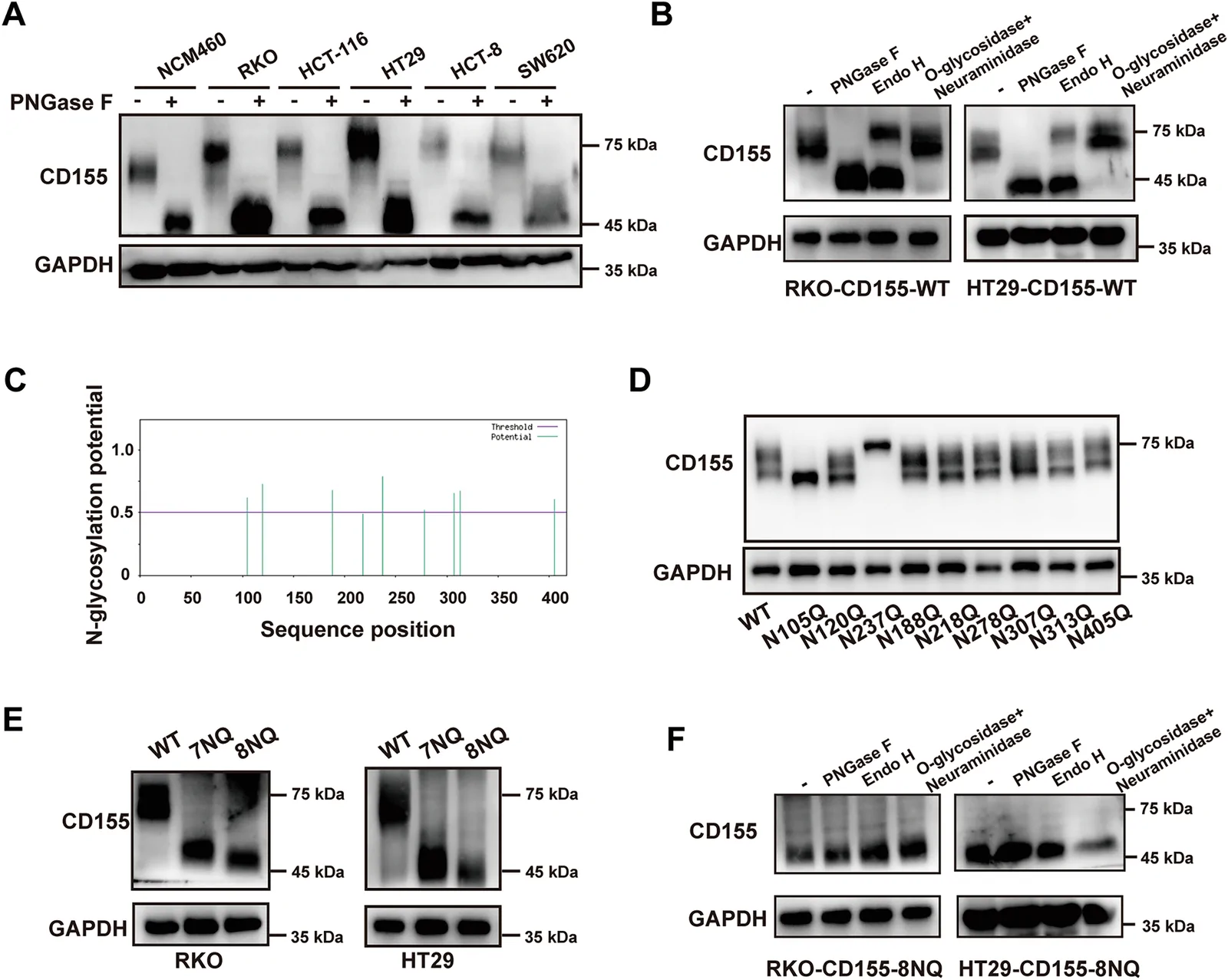
CD155 undergoes N-glycosylation in CRC cell lines. **A** Western blot analysis of CD155 expression in indicated CRC cell lines treated with or without PNGase F. **B** Western blot analysis of CD155 molecular weight shifts in CD155-overexpressing RKO and HT29 cells following treatment with PNGase F, Endo H, or a mixture of O-glycosidase and neuraminidase. **C** Prediction of potential N-glycosylation sites in the CD155 amino acid sequence using NetNGlyc-1.0. **D** Western blot analysis of CD155 expression in CRC cells expressing various CD155 glycosylation site mutants. **E** Western blot analysis of CD155 expression in CRC cells transfected with CD155-7NQ and CD155-8NQ plasmids. **F** Western blot analysis of CD155 expression in CD155-8NQ-expressing CRC cells following treatment with PNGase F, Endo H, or a mixture of O-glycosidase and neuraminidase.

While examining CD155 protein expression across several colorectal cell lines, we detected CD155 as a major band at ~70 kDa, which is markedly higher than its predicted molecular weight of 45 kDa (Fig. 1A). This discrepancy suggested that CD155 may undergo post-translational modification(s). To further explore the functional role of CD155, we selected the RKO and HT29 cell lines for subsequent experiments, both of which exhibited relatively high CD155 expression levels (Fig. 1A). To minimize interference from endogenous CD155, stable CD155-knockout RKO and HT29 cell lines were generated (Supplementary Fig. S1A) and then reconstituted with plasmids encoding wild-type (WT) CD155.

Given that protein glycosylation typically increases the apparent molecular weight on immunoblotting, we next investigated whether glycosylation contributes to the size shift of CD155. Treatment of HT29 and RKO cells with peptide-N-glycosidase F (PNGase F), which removes nearly all N-linked glycans, resulted in a complete shift of CD155 from ~70 kDa to ~45 kDa in both cell lines (Fig. 1A), indicating that N-glycosylation is primarily responsible for the increased molecular weight of CD155. In contrast, treatment with endoglycosidase-H (Endo H), which selectively removes high-mannose and some hybrid *N*-glycans but not mature complex *N*-glycans, caused only a partial reduction in CD155 molecular weight (Fig. 1B). We also treated the samples with neuraminidase, which removes terminal sialic acid residues and thereby facilitates the cleavage of certain O-linked glycans by O-glycosidase. Combined neuraminidase and O-glycosidase treatment did not cause an obvious mobility shift of CD155 (Fig. 1B). Together, these results suggest that the higher molecular weight of CD155 is attributable to glycosylation, with N-linked glycans serving as the predominant modification.

To pinpoint the specific glycosylation sites on CD155, we analyzed the human CD155 amino acid sequence using the NetNGlyc-1.0 server, which predicted nine putative N-glycosylation motifs (Fig. 1C). We then individually mutated candidate asparagine residues to glutamine and assessed the molecular weight of each mutant by immunoblotting. Compared with WT CD155, most single-site mutants, including N105Q, N120Q, N188Q, N218Q, N278Q, N307Q, and N313Q, exhibited varying degrees of molecular weight reduction, whereas the N237Q and N405Q mutant did not show an obvious mobility change (Fig. 1D). These findings indicate that multiple predicted asparagine residues contribute to CD155 glycosylation (Fig. 1D).

To further determine the overall contribution of these sites, we generated multiple-site mutants in which seven key asparagine residues (N105, N120, N188, N218, N278, N307, and N313) were simultaneously substituted with glutamine (7NQ) or, additionally, including N237 to generate an eight-site mutant (8NQ). Both 7NQ- and 8NQ-reconstituted cells exhibited a marked decrease in the apparent molecular weight of CD155 compared with WT, consistent with substantial loss of glycosylation (Fig. 1E). Notably, the 8NQ mutant exhibited a lower molecular weight than the 7NQ mutant (Fig. 1E), suggesting that although mutation of N237 alone did not produce an obvious band shift, this residue may still contribute to CD155 glycosylation in the context of multi-site glycan occupancy. To verify whether residual glycosylation remained in the multi-site mutant, 8NQ-expressing cells were further treated with PNGase F, Endo H, or O-glycosidase and neuraminidase. None of these enzymes induced an additional reduction in the molecular weight of CD155 (Fig. 1F). Collectively, these results establish that CD155 in CRC cells is extensively modified by N-linked glycosylation at multiple asparagine residues, and that these identified sites account for essentially all detectable glycan-dependent molecular weight shifts.

### N-glycosylation alters the stability and subcellular localization of CD155

**Fig. 2:**
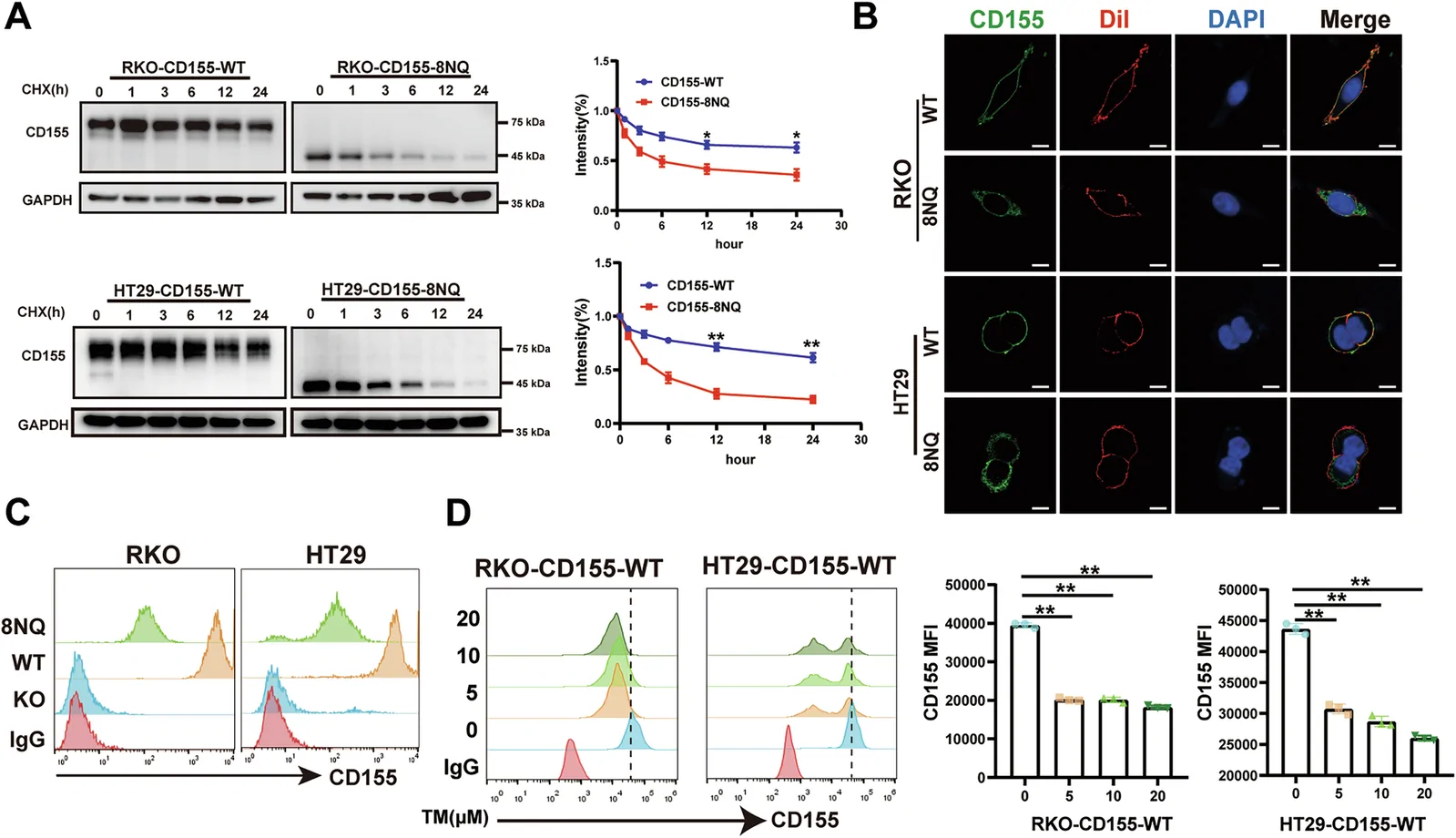
N-glycosylation is essential for the stability and membrane translocation of CD155. **A** Protein stability assay of CD155-WT and CD155-8NQ in RKO and HT29 cells. Cells were treated with cycloheximide (CHX, 20 μmol/L) and harvested at the indicated time points (0, 1, 3, 6, 12, and 24 h). CD155 levels were determined by western blotting (left) and quantified after normalization to GAPDH, with the 0 h value set as 1 (right). **B** Representative immunofluorescence images showing the spatial distribution of CD155 (green), Dil (red) and DAPI (blue). Scale bars, 5 μm. **C** Flow cytometric analysis of surface CD155 expression in CD155-KO, CD155-WT, and CD155-8NQ RKO and HT29 cells. **D** Flow cytometric analysis of surface CD155 expression in CD155-WT RKO and HT29 cells treated with indicated concentrations of Tunicamycin, with corresponding statistical analysis of Mean Fluorescence Intensity (MFI). The data represent the mean ± SD. *P < 0.05, **P < 0.01, ***P < 0.001.

Since CD155 is heavily glycosylated in CRC cell lines, we next verified whether glycosylation affects CD155 protein stability. Following cycloheximide (CHX) treatment to inhibit protein synthesis, CD155 protein levels decreased in both CD155-WT and CD155-8NQ cells. However, the reduction was more pronounced in CD155-8NQ cells, indicating that loss of glycosylation accelerates CD155 protein turnover (Fig. 2A).

We then investigated whether glycosylation influences the subcellular localization of CD155. Confocal microscopy showed that in CD155-WT cells, CD155 staining was almost completely colocalized with the plasma membrane marker. In contrast, CD155-8NQ mutant cells exhibited a marked reduction in plasma membrane localization, with its signal predominantly redistributed to intracellular compartments (Fig. 2B). Consistent with these imaging findings, flow cytometric analysis further demonstrated that the surface CD155 fluorescence peak in CD155-WT cells was higher than that in CD155-8NQ cells in both RKO and HT29 cell lines (Fig. 2C), indicating reduced cell surface expression of the CD155-8NQ mutant. Moreover, treatment of CD155-WT cells with Tunicamycin (TM), an inhibitor of N-linked glycosylation, led to a marked decrease in CD155 surface abundance (Fig. 2D). Collectively, our data support that glycosylation plays an important role in maintaining CD155 protein stability and promoting its localization to the plasma membrane.

### Disruption of CD155 N-glycosylation sensitizes tumor cells to NK cell-mediated cytotoxicity

**Fig. 3:**
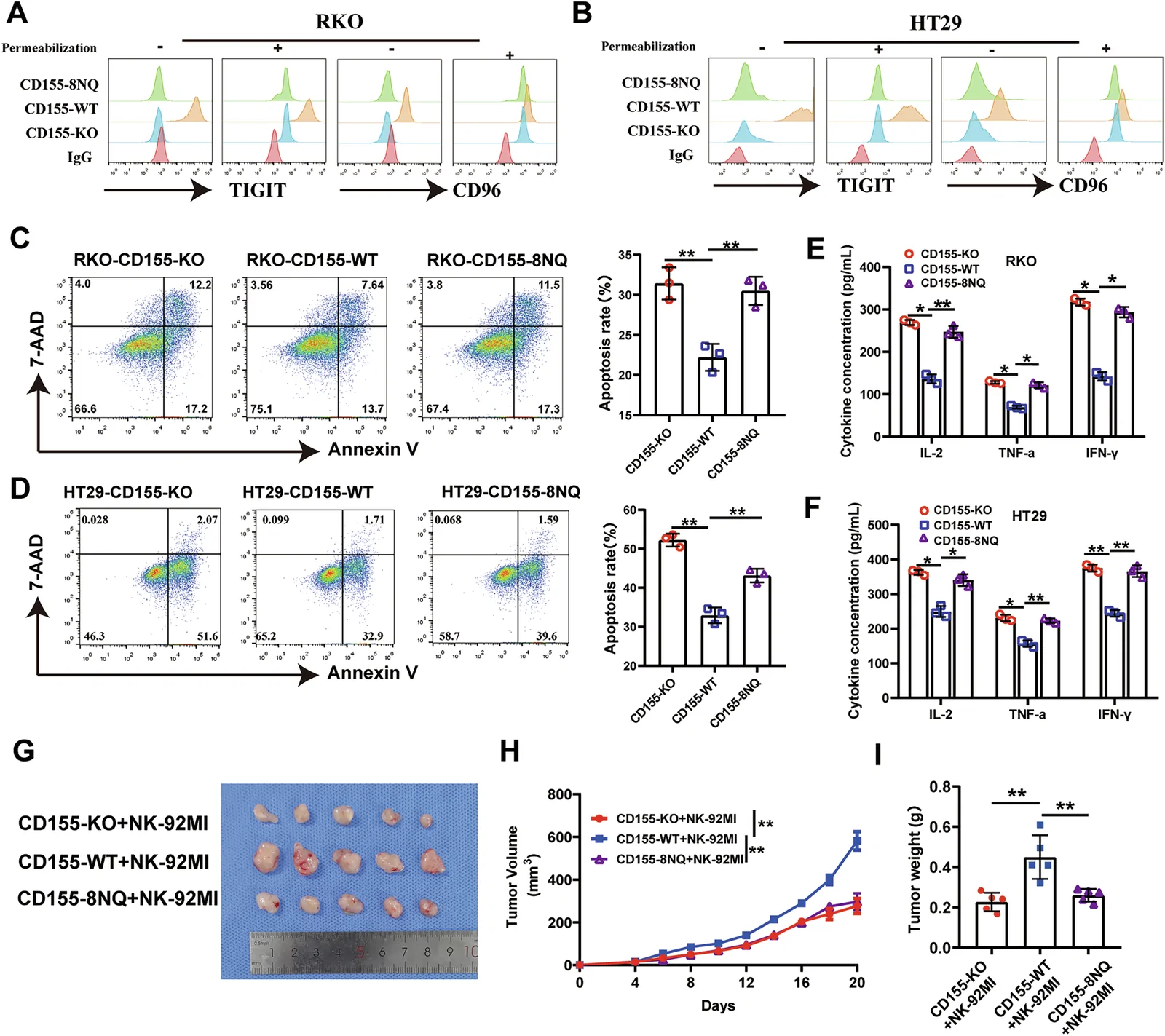
Disruption of CD155 N-glycosylation sensitizes tumor cells to NK cell-mediated cytotoxicity. Flow cytometric analysis of recombinant TIGIT-Fc and CD96-Fc binding to RKO (**A**) and HT29 (**B**) cells (WT, 8NQ, and KO) with or without membrane permeabilization. Flow cytometric analysis and statistical quantification of apoptosis in RKO (**C**) and HT29 (**D**) cells (WT, 8NQ, and KO) following co-culture with NK-92MI cells. IL-2, TNF-α, and IFN-γ levels in the supernatants of RKO (**E**) and HT29 (**F**) cells (WT, 8NQ, and KO) co-cultured with NK-92MI cells. **G** Representative images of subcutaneous tumors from NSG mice inoculated with CD155-KO, CD155-WT, or CD155-8NQ HT29 cells followed by tail vein injection of NK-92MI cells (*n* = 5 per group). Tumor growth curves (**H**) and tumor weights (**I**) derived from the NSG mouse xenograft models described in (**G**). The data represent the mean ± SD. *P < 0.05, **P < 0.01, ***P < 0.001.

To determine whether CD155 glycosylation contributes to tumor cell-mediated immunosuppression, we compared the immunoregulatory activity of CD155 WT, CD155-KO and CD155-8NQ cells both in vitro *and* in vivo. We first confirmed that CD155 knockout or mutation did not markedly affect the intrinsic proliferative capacity of CRC cells in vitro (Supplementary Fig. S2A), thereby excluding the possibility that subsequent functional differences were primarily due to altered tumor cell growth. Because CD155 mediates immune suppression in part through engagement of the inhibitory NK cell receptors TIGIT and CD96, we next assessed whether CD155-8NQ cells differed in the amount of recombinant TIGIT or CD96 bound to the tumor-cell surface. CD155-WT, CD155-8NQ, and CD155-KO tumor cells were incubated with recombinant TIGIT or CD96, and cell-associated receptor binding was quantified by flow cytometry. CD155-WT cells showed robust binding to both TIGIT and CD96. In contrast, TIGIT and CD96 binding to CD155-8NQ cells was reduced and was comparable to that observed in CD155-KO cells (Fig. 3A, B). Similar trends were observed after cell permeabilization (Fig. 3A, B). To determine whether disruption of CD155 N-glycosylation affects tumor-cell susceptibility to NK cell-mediated cytotoxicity, we directly cocultured these tumor cells with NK-92MI cells. CD155-8NQ and CD155-KO tumor cells exhibited comparable levels of apoptosis, both of which were significantly higher than those observed in CD155-WT cells (Fig. 3C, D). Consistently, the levels of IL-2, IFN-γ, and TNF-α were comparable between the CD155-KO and CD155-8NQ groups and were markedly higher than those in the CD155-WT group (Fig. 3E, F). These results suggest that CD155 loss or mutation of its predicted N-glycosylation sites relieves CD155-mediated suppression of NK cell activation and increases tumor cell susceptibility to NK cell killing.

To determine whether TIGIT and CD96 functionally contribute to CD155-dependent NK-cell suppression in our system, we examined their respective contributions by targeting these receptors on NK-92MI cells. Flow cytometry confirmed detectable expression of both TIGIT and CD96, which were effectively reduced upon siRNA-mediated knockdown (Supplementary Fig. S3A, E). In a co-culture system of NK-92MI cells with CD155-WT tumor cells, individual knockdown of either TIGIT or CD96 partially increased the apoptosis of CD155-WT tumor cells (Supplementary Fig. S3B, C, F, G) and enhanced NK-cell activation, as evidenced by elevated levels of IL-2, TNF-α, and IFN-γ in the culture supernatant (Supplementary Fig. S3D, H).

To further validate these findings in vivo, NSG mice bearing CD155-WT, CD155-KO, or CD155-8NQ tumors were treated with NK-92MI cells. In the presence of NK cells, tumors derived from CD155-KO and CD155-8NQ cells grew substantially more slowly than CD155-WT tumors (Fig. 3G–I). Collectively, these results suggest that loss of CD155 or disruption of CD155 N-glycosylation diminishes CD155-mediated inhibitory signaling and increases tumor cell susceptibility to NK cell killing.

### STT3A contributes to the glycosylation of CD155

**Fig. 4:**
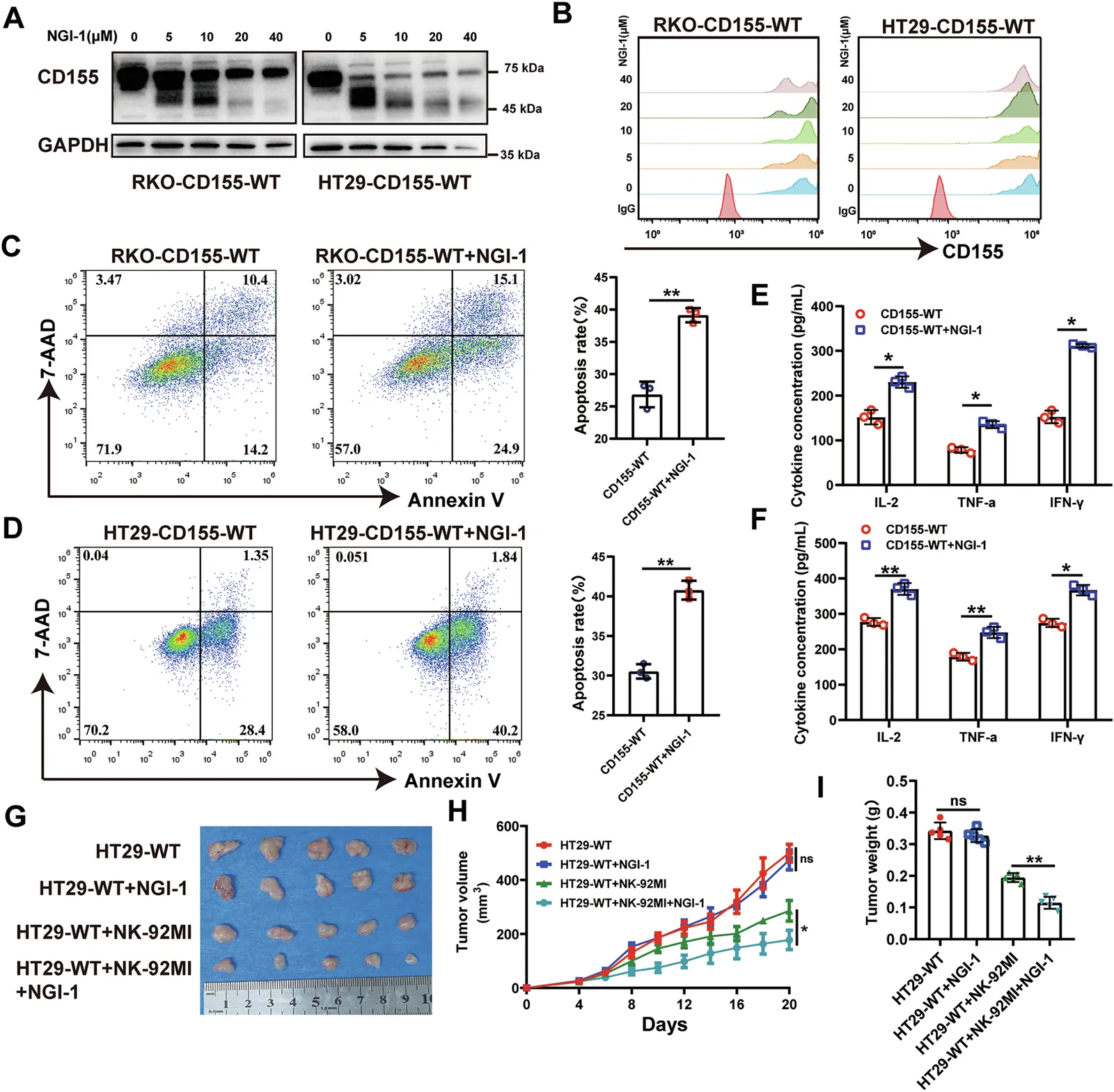
STT3A contributes to the glycosylation of CD155. **A** Western blot analysis of CD155 expression in RKO-CD155-WT and HT29-CD155-WT cells treated with indicated concentrations of NGI-1 (0, 5, 10, and 20 μM) for 24 h. **B** Flow cytometric analysis of surface CD155 expression in RKO-CD155-WT and HT29-CD155-WT cells treated with indicated concentrations of NGI-1 for 24 h. Representative flow cytometry plots and statistical quantification of apoptosis in RKO-CD155-WT (**C**) and HT29-CD155-WT (**D**) cells treated with or without 20 μM NGI-1 followed by co-culture with NK-92MI cells. **E**, **F** IL-2, TNF-α, and IFN-γ levels in the supernatants of RKO-CD155-WT (**E**) and HT29-CD155-WT (**F**) cells treated with or without NGI-1 and co-cultured with NK-92MI cells. **G** Representative images of subcutaneous HT29-WT xenografts in NSG mice following treatment with or without tail vein injection of NK-92MI cells and NGI-1 administration. Tumor growth curves (**H**) and terminal tumor weights (**I**) from the NSG mouse models in the indicated treatment groups (*n* = 5 per group). The data represent the mean ± SD. *P < 0.05, **P < 0.01, ***P < 0.001.

Given that STT3A is a key catalytic component of the OST complex responsible for N-linked glycosylation, we investigated the impact of N-glycosylation on CD155 using the N-glycosylation inhibitor NGI-1 and STT3A knockdown. In CD155-WT RKO and HT29 tumor cells, NGI-1 treatment led to a dose-dependent decrease in both molecular weight and protein abundance of CD155 (Fig. 4A), consistent with impaired N-glycosylation and decreased protein stability. Flow cytometric analysis also showed that NGI-1 treatment markedly reduced membrane CD155 levels in CD155-WT tumor cells (Fig. 4B). Functionally, NGI-1-treated CD155-WT tumor cells exhibited increased apoptosis upon co-culture with NK-92MI cells compared with untreated controls (Fig. 4C, D). Consistently, the levels of IL-2, IFN-γ, and TNF-α were increased following NGI-1 treatment (Fig. 4E, F). To further evaluate the contribution of STT3A, we knocked down STT3A in CD155-WT tumor cells (Supplementary Fig. S4A, B). Similar to pharmacological inhibition of N-glycosylation, STT3A knockdown reduced the apparent molecular weight and protein abundance of CD155 (Supplementary Fig. S4B). Moreover, STT3A knockdown in CD155-WT tumor cells was associated with increased tumor cell apoptosis during co-culture with NK-92MI cells (Supplementary Fig. S4C, D) and elevated levels of IL-2, TNF-α, and IFN-γ in the co-culture supernatants (Supplementary Fig. S4E, F).

We next assessed whether pharmacological inhibition of N-glycosylation by NGI-1 affects NK-cell-mediated antitumor activity in vivo. In NSG mice bearing CD155-WT tumors, NGI-1 treatment alone did not significantly affect tumor growth. However, in the presence of NK cells, NGI-1 administration markedly delayed tumor progression compared with the control group (Fig. 4G–I). Collectively, these results suggest that STT3A-dependent N-glycosylation regulates CD155 expression, thereby influencing NK-cell-mediated tumor cell killing.

### Higher expression of CD155 and STT3A is associated with worse survival

**Fig. 5:**
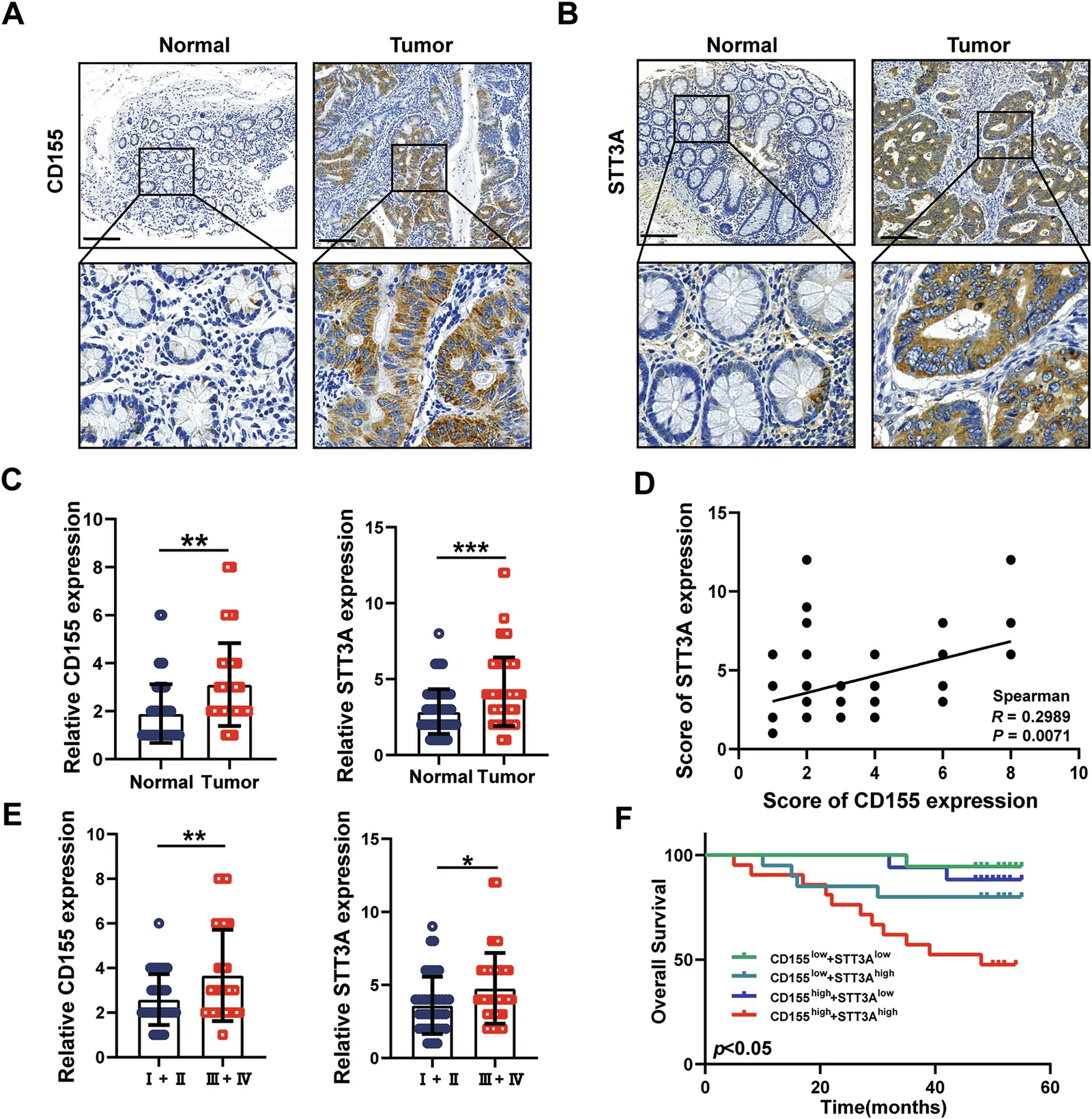
Clinical correlation between CD155 and STT3A expression in CRC patients. **A** Representative IHC images showing CD155 expression in human CRC tissues and paired non-malignant adjacent tissues (*n* = 80). Scale bars, 100 μm. **B** Representative IHC images showing STT3A expression in human CRC tissues and paired non-malignant adjacent tissues (*n* = 80). Scale bars, 100 μm. **C** Statistical quantification of CD155 (left) and STT3A (right) staining indices in human CRC tissues compared to paired non-malignant adjacent tissues. **D** Correlation analysis between the staining indices of CD155 and STT3A in human CRC specimens (*n* = 80). **E** Statistical comparison of CD155 (left) and STT3A (right) staining indices between early-stage (I + II) and late-stage (III + IV) CRC specimens. **F** Kaplan–Meier overall survival curves of CRC patients stratified into four subtypes based on the expression levels of CD155 and STT3A. The data represent the mean ± SD. *P < 0.05, **P < 0.01, ***P < 0.001.

Previous studies have shown that CD155 is upregulated in CRC and is associated with unfavorable clinical outcomes [12, 13]. To directly assess whether CD155 is glycosylated in human CRC tissues, proteins extracted from fresh-frozen tumor tissues were subjected to PNGase F digestion followed by immunoblotting. CD155 from tumor tissues exhibited a higher-molecular-weight smear that shifted to a lower-molecular-weight band after PNGase F treatment, supporting the presence of N-glycosylated CD155 species in clinical CRC samples (Supplementary Fig. S5A). Then we analyzed the expression patterns of CD155 and STT3A in paired primary tumor tissues and matched adjacent normal biopsies from patients with CRC. Immunohistochemistry (IHC) analysis indicated that the protein expression levels of both CD155 and STT3A were significantly higher in tumor tissues than in adjacent normal tissues (Fig. 5A–C). Additionally, CD155 expression was positively associated with STT3A expression in tumor tissues (Fig. 5D). We further compared their levels across different clinical stages. Both CD155 and STT3A expression were significantly higher in patients with advanced-stage disease (stage III + IV) than in those with early-stage disease (stage I + II) (Fig. 5E). Moreover, patients with concurrent high expression of both CD155 and STT3A had significantly worse overall survival than patients with high expression of either marker alone or low expression of both markers (Fig. 5F). These clinical data indicate that CD155 undergoes N-glycosylation in human CRC tissues. Furthermore, elevated expression of CD155 and STT3A is associated with advanced disease and poor prognosis in CRC patients.

## Discussion

**Fig. 6:**
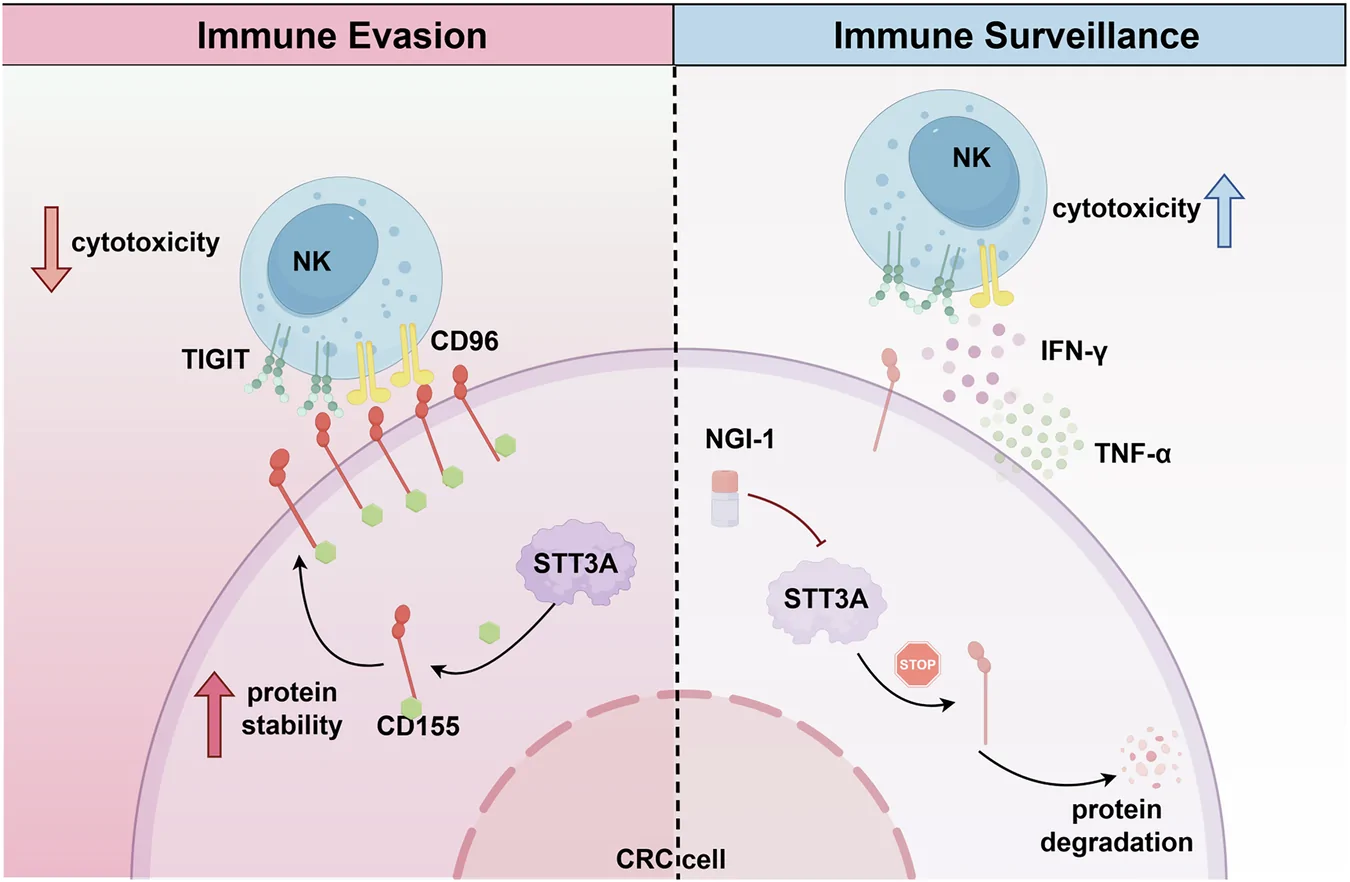
STT3A-mediated CD155 N-glycosylation promotes immune evasion in CRC.

In this study, we dissected a post-translational regulatory mechanism by which CRC cells maintain CD155 protein stability to suppress NK cell-mediated antitumor immunity. We demonstrate that extensive N-linked glycosylation protects CD155 from degradation and sustains its surface expression on tumor cells and STT3A contributes substantially to this modification. CD155 stabilization is driven by a complex multi-site glycosylation network. Disruption of this network profoundly accelerates CD155 turnover. These findings identify STT3A-dependent CD155 N-glycosylation as a critical event that maintains CD155-mediated immunosuppression at the tumor-NK cell interface (Fig. 6).

Prior studies have established that CD155 expression is tightly regulated at the transcriptional level by diverse signaling mechanisms. CCNC has been identified as a transcriptional suppressor of CD155, whereas the kinase DYRK1A functions as a positive regulator [39, 40]. Additionally, CD155 transcription is induced by a broad array of signaling pathways, including the pleiotropic cytokine IL-22 [22], the canonical pro-survival PI3K/Akt and TRAF2–NF-κB cascades [40, 41], and adhesion- and mechano-sensing pathways such as FAK/JNK/c-Jun and RhoA/YAP [15, 42]. However, transcriptional upregulation alone is often insufficient to ensure sustainable surface protein residency. Our findings support a model in which STT3A-dependent N-glycosylation maintains the stability and cell-surface abundance of CD155, thereby increasing its availability for engagement with the CD155-binding NK-cell receptors TIGIT and CD96. These results complement the existing understanding of transcriptional regulation by uncovering a post-translational layer of CD155 control that directly impacts antitumor immunity.

The therapeutic targeting of CD155 and its binding receptors has become a focus of intensive investigation [18, 20, 43]. Leveraging the selective overexpression of CD155 in tumors, direct CD155-targeting modalities, such as oncolytic poliovirus derivatives and CD155-based CAR-T cells, have demonstrated potent antitumor activity in preclinical models [44–47]. Furthermore, antibody-mediated blockade of the CD155-TIGIT axis reshapes the immunosuppressive tumor microenvironment and exhibits substantial antitumor efficacy [43, 48, 49]. CD96 blockade has also shown therapeutic benefit in selected metastatic glioma and melanoma models [50], although the context-dependent inhibitory and costimulatory functions of CD96 complicate its therapeutic development. Because TIGIT is frequently co-expressed with other immune checkpoints such as PD-1 and CTLA-4 on tumor-infiltrating lymphocytes, monotherapy with TIGIT inhibitors has shown limited clinical efficacy [51–53]. Consequently, combination strategies have become a major focus of investigation. Notably, dual blockade of the CD155/TIGIT and PD-1/PD-L1 axis has demonstrated synergistic antitumor activity in preclinical studies [20, 54, 55], and multiple clinical trials are currently evaluating this combination [56]. Encouraging efficacy has been reported with dual TIGIT/PD‑(L)1 inhibition in PD‑L1‑positive non‑small‑cell lung cancer, advanced biliary tract cancer, and HER2‑negative gastroesophageal adenocarcinoma [57–59]. However, the clinical benefit of adding TIGIT blockade appears to be context-dependent: tobemstomig (anti-PD-1/anti-LAG-3 bispecific antibody) plus tiragolumab (anti-TIGIT monoclonal antibody) and atezolizumab plus tiragolumab failed to improve pathological responses compared with nivolumab plus ipilimumab in stage III melanoma [60]. Tumor heterogeneity may underlie the limited efficacy of current combination regimens, highlighting the need to profile CD155 and its receptors on tumor-infiltrating lymphocytes for improved patient stratification.

Our study demonstrates that the N-glycosylation inhibitor NGI-1 dose-dependently inhibits CD155 glycosylation, and that the combination of NGI-1 with NK cell therapy suppresses tumor growth more effectively than either agent alone in vivo. It should be noted that NGI-1 is a broad-spectrum N-glycosylation inhibitor [61], and its antitumor effects cannot be attributed exclusively to CD155. In addition to destabilizing glycosylated CD155, NGI-1 may affect other glycoproteins involved in tumor progression and immune regulation, including immune checkpoint proteins such as PD-L1 [62]. Whether this broad activity translates into improved antitumor efficacy remains to be determined. Moreover, given the essential roles of N-glycosylation in numerous physiological processes, broad inhibition may cause off-target effects and systemic toxicity, which will require careful preclinical evaluation [63]. To circumvent these potential off-target toxicities and achieve more precise therapeutic intervention, future efforts should focus on developing highly specific monoclonal antibodies that selectively target the uniquely glycosylated epitopes of CD155.

Several additional limitations should be acknowledged. First, the CD155-8NQ mutant exhibited reduced protein stability and impaired plasma membrane localization, which resulted in decreased binding to its inhibitory receptors. However, whether N-glycans directly participate in receptor binding or primarily regulate the amount and conformation of CD155 available at the cell surface requires further investigation. Second, a notable complexity in translating these findings to human patients is the existence of splice variants that generate soluble CD155 (sCD155), which fundamentally differs from the murine system [64]. Intriguingly, sCD155 preferentially binds to the activating receptor CD226 rather than the inhibitory TIGIT [64]. The impact of STT3A-mediated glycosylation on sCD155 secretion and stability remains an open question. Future studies are warranted to elucidate precisely how glycosylation modifications modulate the structural conformation and functional properties of different CD155 isoforms. Furthermore, it is crucial to identify the specific factors within the tumor microenvironment that dictate whether the CD155 signaling network skews toward immune activation or immunosuppression.

In summary, using CRC as a model, we have delineated a novel immune evasion mechanism wherein tumor cells hijack STT3A-mediated N-glycosylation to stabilize CD155 and suppress NK cell immunosurveillance. Intercepting this post-translational modification impairs CD155-mediated inhibitory signaling and enhances NK cell antitumor activity. Thus, these findings highlight CD155 N-glycosylation as a potential therapeutic vulnerability and provide a rationale for targeting the STT3A-CD155 axis as a therapeutic strategy to overcome CD155-mediated immunosuppression in CRC and potentially in other malignancies.

## Materials and methods

### Cell culture

The normal human colonic epithelial cell line NCM-460, alongside human CRC cell lines (HCT116, HT29, RKO, HCT8, and SW620) and the NK-92MI cell line, were obtained from the American Type Culture Collection (ATCC, USA). NCM-460 and CRC cells were maintained in DMEM (Eallbio, China), whereas NK-92MI cells were cultured in RPMI-1640 medium (Eallbio). Both culture media were supplemented with 10% fetal bovine serum (FBS; Eallbio) and 1% penicillin-streptomycin (Beyotime, China). All cell lines were incubated in a humidified atmosphere at 37 °C containing 5% CO₂.

### Western blotting

Total protein was isolated from cells using cell lysate (Beyotime) containing protease and phosphatase inhibitors. Protein concentrations were quantified using a BCA Protein Assay Kit (Beyotime). Equal amounts of protein lysates were separated via SDS-PAGE and transferred onto membranes, followed by sequential incubation with primary antibodies against CD155 (1:1,000; CST, USA, #81254T), DYKDDDDK tag (1:10,000; Proteintech, China, #66008-4-Ig), STT3A (1:2000; Proteintech, #66581-1-Ig) and GAPDH (1:20,000; Proteintech, #60004-1-Ig). Finally, the signal was detected using an enhanced chemiluminescence (ECL) detection system, following the manufacturer’s recommended protocol.

### Patient samples

Two tissue microarray (TMA) gene chips were provided by Hunan Aifang Biotechnology Co., Ltd., which included 80 CRC tissue samples and related follow-up data. The patient characteristics are detailed in Supplementary Table S1. This study was approved by the ethics committee for the research protocol (Ethics Number: 2026362).

### Immunohistochemistry (IHC)

Formalin-fixed, paraffin-embedded tissue blocks were sectioned at a thickness of 5 μm. Following deparaffinization, rehydration, and heat-induced antigen retrieval, endogenous peroxidases and non-specific binding sites were blocked. Sections were then incubated overnight at 4 °C with primary antibodies against CD155 or STT3A. Subsequently, the slides were incubated with HRP-conjugated secondary antibodies, developed utilizing a 3,3’-diaminobenzidine (DAB) substrate, and observed under a light microscope. Immunoreactivity was semi-quantitatively assessed based on staining intensity (0, negative; 1, weak; 2, moderate; 3, strong) and the percentage of positive cells (1, <25%; 2, 25–50%; 3, 51–75%; 4, >75%).

### CRISPR/Cas9-mediated gene disruption

To generate CD155-deficient cell models, specific CRISPR/Cas9 knockout (KO) plasmids targeting human CD155 were acquired from Miaolingbio (China). Transient transfection of these KO plasmids into RKO and HT29 cell lines was executed utilizing lipofectamine 2000 (Invitrogen, USA). Forty-eight hours post-transfection, cells were harvested and seeded into multiple well plates at a single-cell density to isolate monoclonal populations. Successful ablation of CD155 in the selected clones was strictly validated via immunoblot analysis.

### Plasmid construction and stable transfection

Full-length wild-type human CD155 expression plasmids were sourced from Miaolingbio. Site-directed mutagenesis to generate CD155 mutant constructs was achieved utilizing the ClonExpress II One Step Cloning Kit (Vazyme, China) with specifically designed mutagenic primers. For stable overexpression, lentiviral particles were generated by co-transfecting the target plasmids with packaging (psPAX2) and envelope (pMD2.G) vectors into HEK-293T cells using Lipofectamine 3000 (Invitrogen). RKO and HT29 cells were subsequently transduced with the collected viral supernatants in the presence of polybrene.

### Flow cytometry analysis

Single-cell suspensions were immunolabeled with specific fluorochrome-conjugated monoclonal antibodies and analyzed via multicolor flow cytometry. Isotype-matched antibodies served as negative controls to calibrate background fluorescence. Specifically, Alexa Fluor® 488-anti-CD155 (Biolegend) and Alexa Fluor® 647-anti-His Tag (Biolegend) were employed for surface marker detection. Apoptotic events were quantified using an Annexin V-PE Apoptosis Detection Kit (BD Biosciences). To evaluate cytokine secretion profiles, supernatants from NK-92MI and CRC cell co-cultures were harvested and analyzed utilizing the Human Th1/Th2/Th17 Cytometric Bead Array (CBA) Kit (BD Biosciences) according to the manufacturer’s protocol. Data acquisition was performed on a flow cytometer (Beckman-Coulter), and subsequent analysis was conducted utilizing FlowJo software (version 10.8).

### NK-92MI cell in vitro killing assay

The cytotoxic capability of NK-92MI cells was evaluated utilizing a flow cytometry-based co-culture system. Target CRC cells were pre-stained with carboxyfluorescein diacetate succinimidyl ester (CFSE; YEASEN, China). Following a 6-hour co-incubation with NK-92MI effector cells, the apoptosis rate of the CFSE-positive tumor population was determined via Annexin V-PE staining, strictly adhering to the kit’s guidelines.

### Characterization of CD155 N-glycosylation

To characterize the glycosylation modifications of CD155, cells were harvested in IP lysis buffer. Following the addition of Glycoprotein Denaturing Buffer (10X) and denaturation at 100 °C for 10 min, cell lysates were subjected to enzymatic digestion using PNGase F, Endo H, O-Glycosidase, or Neuraminidase (all from New England Biolabs) under recommended buffer conditions. After overnight incubation at 37 °C, the samples were subjected to immunoblotting. Further, parallel CRC cells were pharmacologically treated with the N-linked glycosylation inhibitor Tunicamycin for 24 h prior to related analyses.

### Binding affinity assay of CD96/TIGIT to CD155

The interactions between CD155 and its respective immune checkpoints were assessed via flow cytometry. CRC cell suspensions were grouped and incubated with pre-formed protein-antibody complexes. Briefly, a mixture of Alexa Fluor® 647-anti-His Tag antibody (10 µL, 10 μg/mL) combined with either His-tagged CD96 protein (5 μg/mL) or His-tagged TIGIT protein (5 μg/mL) was formulated and added to the cell suspensions. Cells treated with equal volumes of PBS and Alexa Fluor® 647-anti-His Tag antibody served as negative controls. The binding capacities of CD96 and TIGIT to the cell surface were quantified based on the fluorescent signal intensity.

### Immunofluorescence staining

For morphological observation, cells from distinct experimental groups were seeded in 24-well plates and properly fixed. Target proteins were probed with a DYKDDDDK Tag rabbit mAb (Proteintech) overnight at 4 °C. On the following day, cells were incubated with corresponding fluorophore-conjugated secondary antibodies, co-stained with the membrane dye DiL (Beyotime) and the nuclear stain DAPI (Beyotime), and ultimately visualized utilizing a confocal laser scanning microscope.

### Cell transfection

For transient gene silencing, NK‑92MI and CRC cells were seeded in 6‑well plates and transfected with Lipofectamine 3000 (Invitrogen, USA) according to the manufacturer’s instructions. NK‑92MI cells were transfected with three independent siRNAs targeting TIGIT or CD96 (siRNA‑1, ‑2, and ‑3) or a negative control siRNA (si‑NC). CRC cells were transfected with three independent siRNAs targeting STT3A (siRNA‑1, ‑2, and ‑3) or si‑NC. Cells were harvested 72 h after transfection, and knockdown efficiency was confirmed by RT‑qPCR and/or flow cytometry prior to subsequent functional experiments. All siRNA sequences are provided in Supplementary Tables S2–S4.

### Quantitative real-time PCR

Total messenger RNA (mRNA) was extracted from cells using TRIZOL reagent (Vazyme) according to the manufacturer’s instructions. Quantitative real‑time PCR was performed with SYBR Green master mix (Roche, USA) in a High Throughput Quantitative PCR LightCycler 480 (Roche). The comparative threshold cycle method was utilized to calculate differences in gene transcription (fold change) by normalization to the reference gene *GAPDH*. Primer sequences for *STT3A* and *GAPDH* are provided in Supplementary Table S5.

### In vivo xenograft tumor models

Female NSG mice (6 weeks old) were procured from the Laboratory Animal Center of Soochow University. To establish xenograft models, 2 × 10⁶ cells of various HT29 variants (HT29-KO, HT29-CD155-WT, or HT29-CD155-8NQ) were inoculated subcutaneously into the flanks of mice. Tumor dimensions were closely monitored every two days starting from day 10 post-inoculation. To further investigate the regulatory role of CD155 N-glycosylation on NK-92MI cell-mediated immune responses, mice bearing the aforementioned xenografts received intravenous infusions of 1 × 10⁷ NK-92MI cells. For in vivo therapeutic evaluation targeting N-glycosylation, mice bearing HT29-CD155-WT tumors were randomized into distinct treatment groups and administered with NGI-1 (20 mg/kg, i.v.). All animal husbandry procedures and in vivo experimental protocols were approved by the Ethics Committee of Soochow University.

### Statistical analyses

All statistical analyses were executed utilizing GraphPad Prism 8.0 software (La Jolla, CA, USA). Differences between two independent groups were evaluated via an unpaired two-tailed Student’s *t* test. For comparisons involving three or more groups, a one-way ANOVA was utilized, while a two-way ANOVA was conducted when assessing two categorical factors. Data are presented as the mean ± SD, and the threshold for statistical significance was set at *P* < 0.05.

## Supplementary information

Supplementary Table S1

Supplementary Table S2

Supplementary Table S3

Supplementary Table S4

Supplementary Table S5

Supplementary Figure S1

Supplementary Figure S2

Supplementary Figure S3

Supplementary Figure S4

Supplementary Figure S5

Supplementary material

Supplementary material-Western blot

## Data Availability

The authors declare that all data generated or analyzed during this study are included in this published article. The data presented in this study are available on request from the corresponding authors.
