# Supplementary material for: N-glycosylation stabilizes CD155 to evade NK cell surveillance in colorectal cancer
Source: Cell Death Discov. 2026 Aug 13;12:449. doi: 10.1038/s41420-026-03302-9 (PMC13748613; doi:10.1038/s41420-026-03302-9)
Supplement: Supplementary file 1 [file 41420_2026_3302_MOESM11_ESM.docx]

**Figure. S1: Validation of CD155 knockout.**

A: Western blot analysis of CD155 expression in RKO and HT29 cells following negative control (NC) treatment or CD155 knockout (KO).

**Figure. S2: Verify the effect of CD155 on colony formation in CRC cells.**

A: Representative images and statistical quantification of colony formation assays in RKO and HT29 cells across NC, KO, and 8NQ groups. Data are representative of three independent experiments. *P* values were calculated using one-way ANOVA, and data are presented as mean ± SD.

**Figure. S3: Silencing TIGIT or CD96 in NK cells enhances their antitumor activity against CD155-expressing CRC cells.**

A: Flow-cytometric analysis of cell-surface TIGIT expression in NK-92MI cells transfected with a negative-control siRNA (si-NC) or three independent TIGIT-targeting siRNAs. An IgG isotype control was included. si-TIGIT-3 was used in subsequent experiments.

B, C: Representative flow-cytometry plots (B) and quantification of the total apoptotic rate (C) of RKO-CD155-WT and HT29-CD155-WT cells following co-culture with NK-92MI cells transfected with si-NC or si-TIGIT-3.

D: IL-2, TNF-α, and IFN-γ concentrations in the supernatants of RKO-CD155-WT (left) and HT29-CD155-WT (right) cells co-cultured with NK-92MI cells transfected with si-NC or si-TIGIT-3.

E: Flow-cytometric analysis of cell-surface CD96 expression in NK-92MI cells transfected with si-NC or three independent CD96-targeting siRNAs. An IgG isotype control was included. si-CD96-1 was used in subsequent experiments.

F, G: Representative flow-cytometry plots (F) and quantification of the total apoptotic rate (G) of RKO-CD155-WT and HT29-CD155-WT cells following co-culture with NK-92MI cells transfected with si-NC or si-CD96-1.

H: IL-2, TNF-α, and IFN-γ concentrations in the supernatants of RKO-CD155-WT (left) and HT29-CD155-WT (right) cells co-cultured with NK-92MI cells transfected with si-NC or si-CD96-1. A-H, Data are representative of three independent experiments and presented as the mean ± SD. **P* < 0.05, ***P* < 0.01, ****P* < 0.001. *P* values were calculated using two-tailed unpaired Student’s t-tests (C, D, G, H).

**Figure. S4: STT3A silencing inhibits CD155 glycosylation and sensitizes CRC cells to NK cell cytotoxicity.**

A: RT-qPCR analysis of STT3A mRNA expression in RKO-CD155-WT and HT29-CD155-WT cells transfected with negative-control siRNA (si-NC) or three independent STT3A-targeting siRNAs.

B: Western blot analysis of CD155 and STT3A protein expression in RKO-CD155-WT and HT29-CD155-WT cells following transfection with si-NC or the indicated STT3A-targeting siRNAs.

C, D: Representative flow-cytometry plots (left) and quantification (right) of the total apoptotic rate of si-NC or si-STT3A-transfected RKO-CD155-WT (C) and HT29-CD155-WT (D) cells following co-culture with NK-92MI cells.

E, F: Concentrations of IL-2, TNF-α, and IFN-γ in the supernatants of NK-92MI cells co-cultured with si-NC or si-STT3A-transfected RKO-CD155-WT (E) and HT29-CD155-WT (F) cells. A-F, Data are representative of three independent experiments and presented as the mean ± SD. **P* < 0.05, ***P* < 0.01, ****P* < 0.001. *P* values were calculated using two-tailed unpaired Student’s t-tests (C-F) or one-way ANOVA (A).

**Figure. S5: CD155 is glycosylated in human tumor tissues.**

A: Western blot analysis of CD155 expression in protein lysates from fresh-frozen CRC tumor tissues, with or without PNGase F treatment.
