# Supplementary material for: N-glycosylation stabilizes CD155 to evade NK cell surveillance in colorectal cancer
Source: Cell Death Discov. 2026 Aug 13;12:449. doi: 10.1038/s41420-026-03302-9 (PMC13748613; doi:10.1038/s41420-026-03302-9)
Supplement: Supplementary file 2 [file 41420_2026_3302_MOESM12_ESM.docx]

Figure1:

A:

GAPDH	CD155

B

:

D:

E:

Fig2A:

Fig4A:
