# Supplementary material for: N-glycosylation stabilizes CD155 to evade NK cell surveillance in colorectal cancer
Source: Cell Death Discov. 2026 Aug 13;12:449. doi: 10.1038/s41420-026-03302-9 (PMC13748613; doi:10.1038/s41420-026-03302-9)
Supplement: Supplementary file 3 [file 41420_2026_3302_MOESM1_ESM.docx]

| **Table S1. Correlation between CD155 expression and clinical features in patients with CRC(n= 80).** | | | | |
| --- | --- | --- | --- | --- |
| **Characteristic Total NO.** | | **CD155 expression** |  | **P value** |
|  |  | **High** | **Low** |  |
| **All case** | 80 | 42 | 38 |  |
| **Age** |  |  |  | 0.6378 |
| >60 | 42 | 21 | 21 |  |
| ≤60 | 38 | 21 | 17 |  |
| **Gender** |  |  |  | 0.1919 |
| Male | 44 | 26 | 18 |  |
| Female | 36 | 16 | 20 |  |
| **Tumor depth** |  |  |  |  |
| T1/T2 | 8 | 5 | 3 | 0.5505 |
| T3/T4 | 72 | 37 | 35 |  |
| **LN involvement** |  |  |  |  |
| N0 | 46 | 22 | 24 | 0.3302 |
| N1/2 | 34 | 20 | 14 |  |
| **Metastasis** |  |  |  |  |
| M0 | 69 | 34 | 35 | 0.148 |
| M1 | 11 | 8 | 3 |  |
