# Supplementary material for: N-glycosylation stabilizes CD155 to evade NK cell surveillance in colorectal cancer
Source: Cell Death Discov. 2026 Aug 13;12:449. doi: 10.1038/s41420-026-03302-9 (PMC13748613; doi:10.1038/s41420-026-03302-9)
Supplement: Supplementary file 6 [file 41420_2026_3302_MOESM4_ESM.docx]

| **Table S4. Target sequence of CD96 siRNA** | | |
| --- | --- | --- |
| **si-RNA** |  | **Primer sequence** |
| si-CD96-1 |  | GGAAAUAAAGUGUGGAACATT  UGUUCCACACUUUAUUUCCTT |
| si-CD96-2 |  | GCAAAUAUCACAUGGUUUATT  UAAACCAUGUGAUAUUUGCTT |
| si-CD96-3 |  | UAACAAGGGUACAUAGUAATT  UUACUAUGUACCCUUGUUATT |
