# Supplementary material for: N-glycosylation stabilizes CD155 to evade NK cell surveillance in colorectal cancer
Source: Cell Death Discov. 2026 Aug 13;12:449. doi: 10.1038/s41420-026-03302-9 (PMC13748613; doi:10.1038/s41420-026-03302-9)
Supplement: Supplementary file 7 [file 41420_2026_3302_MOESM5_ESM.docx]

| **Table S5. Primers for RT-qPCR** | | |
| --- | --- | --- |
| **Primer Name Primer sequence** | | |
| hsa-GAPDH-Forward |  | GGAGCGAGATCCCTCCAAAAT |
| hsa-GAPDH-Reverse |  | GGCTGTTGTCATACTTCTCATGG |
| hsa-STT3A-Forward |  | TTGGGACGAATCATTGGAGGA |
| hsa-STT3A-Reverse |  | GTAAGGTGGTACGTGACGATG |
